# Growth of Li_*x*_La_*y*_Sr_*z*_MnO_3_ thin films by pulsed laser deposition: complex relation between thin film composition and deposition parameters

**DOI:** 10.1007/s00339-021-04506-9

**Published:** 2021-05-31

**Authors:** G. Bimashofer, S. Smetaczek, E. Gilardi, C. W. Schneider, A. Limbeck, T. Lippert, J. Stahn

**Affiliations:** 1 Paul Scherrer Institute, 5232 Villigen, Switzerland; 2 Department of Chemistry and Applied Biosciences ETH Zürich, 8093 Zürich, Switzerland; 3 Institute of Chemical Technologies and Analytics, TU Wien, 1060 Vienna, Austria

**Keywords:** Thin film, Pulsed laser deposition, Complex oxide, Lithium

## Abstract

Li_*x*_La_*y*_Sr_*z*_MnO_3_ thin films of various compositions (*x*,*y*,*z*) have been grown using pulsed laser deposition. The compositions of the films have been studied as a function of deposition temperature, target-to-substrate distance and deposition pressure with respect to different cation ratios of the targets by inductively coupled plasma mass spectrometry. When growing multi-elemental oxide thin films containing lithium (with its large mass difference to other elements), lithium loss is most probably inevitable. But the desired thin film composition can be achieved by selecting specific growth conditions and different target compositions. The experiments also elucidate some of the mechanisms behind the incongruent lithium transfer from the targets to thin films.

## Introduction

The general interest to fabricate lithium containing thin films has been growing since they can be used and integrated in micro-batteries utilised as power sources for nano-devices like smart cards, implantable medical devices and microsensors. [1, 2] For these applications, it is necessary to grow high-quality thin films (with respect to crystallinity, composition, etc.) containing light and reactive elements like Li and heavier elements such as Co, Mn, Ni. [3] The increased activity in developing all solid-state lithium batteries has therefore sparked extensive research to produce and characterise lithium containing films including oxides. [4, 5]

Our future aim is to electrochemically control the magnetic properties of lithiated La_1−*x*_Sr_*x*_MnO_3_ (LSMO) to help disentangle the relationship between charge ordering and magnetism in manganites. The magnetic properties of LSMO are a function of the doping level and hence of the Mn oxidation state. [6] Introducing Li into the overall composition will contribute to the net valence and therefore provides means to study properties of magnetic phase boundaries in systems like La_1−*x*_Sr_*x*_MnO_3_ with *x* $\approx$ 0.5, where this material exhibits a transition between ferro- and paramagnetism at room temperature. [7]

Pulsed laser deposition (PLD) is widely used to fabricate thin oxide films with a complex composition because of the often described congruent material transfer. [8] Conversely, it has been shown that a congruent transfer is not always the case and depends on a wide range of parameters. [9] One very important parameter is the mass ratio of the involved species with a large ratio leading to distinct composition deficiencies. [10] Several studies exhibited the influence of the used background gas on the composition of the grown film. [8, 10–14] E.g. Ohnishi et al. [15] proved that LiCoO_2_ films become lithium deficient at higher oxygen background pressures. For LiMn_2_O_4_, Dumont et al. [16] also showed that there are deviations in composition for high background pressures and therefore have a strong influence on chemical and physical properties. From these observations, one can conclude that the fabrication of materials containing a mixture of light and heavy elements with PLD leads to deviations in the films composition at certain pressures.

It is imperative for the magnetic properties of the material to grow highly crystalline LSM/LiLSM thin films with the corresponding oxygen stoichiometry.[17, 18] Therefore, adversely to the warnings in literature, high oxygen background pressures and post annealing are used to grow the films during the conducted experiments.

The focus of this study is to grow lithiated La_1−*x*_Sr_*x*_MnO_3_ (LiLSM) films using PLD from ceramic targets with variations in the A-site cation stoichiometries and to establish sample growth parameters for lithiated LSM films, namely Li_0.1_La_0.5_Sr_0.4_MnO_3_ with oxygen as background gas. First, a variety of target compositions was used and process parameters such as background pressure, laser fluence, target-to-substrate distance and temperature were kept constant. In a second series of experiments, two target distances were tested for one target composition (4 cm and 7 cm). Finally, the influence of the deposition temperature upon Li transfer was studied complemented by depositions at room temperature.

PLD was chosen because it allows a high degree of flexibility which might be necessary when trying to optimise growth parameters for multi-element oxides. It also allows the growth of thin films with compositions that are non-existent in bulk by tuning the process parameters. [19] As the method exhibits a high degree of freedom when it comes to growth conditions it is also challenging to find the best conditions for the desired thin film. In this specific case, these experiments were therefore helpful to elucidate some of the dynamics leading to lithium loss during the PLD process when growing multi-element oxide thin films with a mix of very light and heavy cations.

## Experimental details

### Target preparation

Various LiLSM PLD-targets were produced using the solid-state route. For which La_2_O_3_ (Sigma Aldrich 99.999%), SrCO_3_ (Alfa Aesar 99.99%), MnO_2_ (Sigma Aldrich 99%) powders were used in stoichiometric amounts and Li_2_CO_3_ (Alfa Aesar 99%) was applied in excess (10%, 30%, 200%, 400%), to compensate for lithium loss during sintering. [20] The mixtures were pressed into pellets, sintered at 1473K and reground twice. The target-IDs (T for target) correspond to the remaining lithium content determined by inductively coupled plasma mass spectrometry (ICP-MS) in the target after sintering (see Table 1).

**Table 1:** Overview of the as prepared targets, including the lithium content per formula unit (pfu) (relative to Mn=1) before and after sintering

Target-ID	Li content [pfu] ± $\sigma$	
	Before/	After sintering
T-Li-0.33	0.40	0.33 ± 0.0(06)
T-Li-0.13	0.20	0.13 ± 0.00(3)
T-Li-0.08	0.13	0.08 ± 0.01(0)
T-Li-0.06	0.10	0.06 ± 0.00(1)

### Thin film preparation

After pre-ablation of the targets, LiLSM thin films were deposited on $5 \times 5 \times 1$ mm$^{3}$ Yttria-stabilised ZrO_2_ substrates (Crystec). The depositions were performed using an excimer laser ($\lambda$ = 248 nm), ablating four of the ceramic pellets mentioned above, namely T-Li-0.33, T-Li-0.13, T-Li-0.08, T-Li-0.06. The spot size of the laser on target was 1 mm^2^ and the fluence was 1.5 J/cm^2^. A laser frequency of 2 Hz and a substrate temperature of 923K were used, except for an additional deposition of T-Li-0.06 at 823 K. The oxygen background pressure was set between 10 and 50 Pa. This pressure range was chosen to insure the oxygen stoichiometry, and therefore the appropriate Mn oxidation state [21] in the films. The distance from target to substrate was set to 4 cm for most films, one additional ablation sequence with T-Li-0.13 was done at 7 cm with a deposition time of 30 minutes for each film. The subsequent annealing in O_2_ (10^4^ Pa) was done at 723K. The film-IDs (F for film) correspond to the lithium content in the target and the lithium content in the film (Table 2).

**Table 2:** Variation of the process parameters such as temperature *T*, distance *D*, pressure *P* during PLD and the lithium content (pfu) (again relative to Mn=1) in the target and resulting thin film

Target-ID	Film-ID	Process parameters			Li content [pfu] ± $\sigma$	
		*T* [K]	*D* [cm]	*p* [Pa]	Target	Film
T-Li-0.33	F-Li-0.33-0.15	923	4	20	0.33 ± 0.00(6)	0.15 ± 0.02(3)
T-Li-0.13	F-Li-0.13-0.01	923	4	20	0.13 ± 0.00(3)	0.01 ± 0.00(4)
	F-Li-0.13-0.03	923	4	50		0.03 ± 0.01(1)
	F-Li-0.13-0.10	923	7	20		0.10 ± 0.00(5)
T-Li-0.08	F-Li-0.08-0.03	923	4	20	0.08 ± 0.01(0)	0.03 ± 0.0(09)
T-Li-0.06	F-Li-0.06-0.02	923	4	20	0.06 ± 0.00(1)	0.02 ± 0.0(36)
	F-Li-0.06-0.01	823	4	20		0.01 ± 0.00(2)
T-Li-0.33	F-Li-0.33-0.09-rt	RT	4	20	0.33 ±0.0(06)	0.09 ± 0.00(1)
T-Li-0.13	F-Li-0.13-0.04-rt	RT	4	20	0.13 ±0.00(3)	0.04 ± 0.00(2)
T-Li-0.08	F-Li-0.08-0.05-rt	RT	4	20	0.08 ± 0.01(0)	0.05 ± 0.00(4)
T-Li-0.06	F-Li-0.06-0.04-rt	RT	4	20	0.06 ± 0.00(1)	0.04 ± 0.01(4)

## Characterisation

The compositions of the synthesised targets as well as the compositions of the thin films (3 from each target) were determined by ICP-MS. [22] A quadrupole instrument (Thermo iCAP Qc, ThermoFisher Scientific, Germany) was used for all measurements. Sample digestion was achieved using hot aqua regia (80 °C, 3 h). For signal quantification, conventional external calibration using aqueous standards was performed and internal standardisation (1 ng/g indium) was applied. All standards were prepared from certified single element ICP-standard solutions (Certipur, Merck, Germany). Prior to each experiment, instrument settings were optimised for maximum indium-115 signal using a tuning solution provided by the manufacturer of the instrument.

For the crystallographic measurements of the target powders, a high-resolution X-ray powder diffractometer (Bruker D8) with Cu K$\alpha$-radiation ($\lambda$ = 1.54 Å) and Bragg-Brentano geometry was used. Phase identification was done by using the FullProf [23] software. Epitaxy and thickness of the films were measured with a high-resolution X-ray diffractometer (Seifert, $\lambda$ = 1.54 Å). To prove crystallinity of the thin films, rocking curves (for substrate off-set) and $\Theta /2\Theta$  measurements were carried out. For film-thickness calibration, X-ray reflectometry curves were recorded and fitted with GenX. [24] Atomic force microscopy (AFM, Nanosurf FlexAFM, operating in tapping mode) was used to determine surface topography and roughness of the as-deposited films. AFM image processing was done using the Gwyddion [25] software.

## Results and discussion

### Targets

Qualitative phase analysis with the FullProf software revealed that all LiLSM targets contain LiMn_2_O_4_ [26] and ${\mathrm{La}}_{0.5}{\mathrm{Sr}}_{0.5}{\mathrm{MnO}}_{3}$ [27] phases except T-Li-0.33, which contains ${\mathrm{LiLa}}_{3}Sr{\mathrm{MnO}}_{8}$ [28], ${\mathrm{La}}_{0.5}{\mathrm{Sr}}_{0.5}{\mathrm{MnO}}_{3}$ and ${\mathrm{La}}_{0.7}{\mathrm{Sr}}_{0.3}{\mathrm{MnO}}_{2.8}$. [29] Additionally all pellets contain some impurities from the starting oxides (e.g. SrMnO_3_ [30]).

### Thin films

#### Structure

**Fig. 1:**
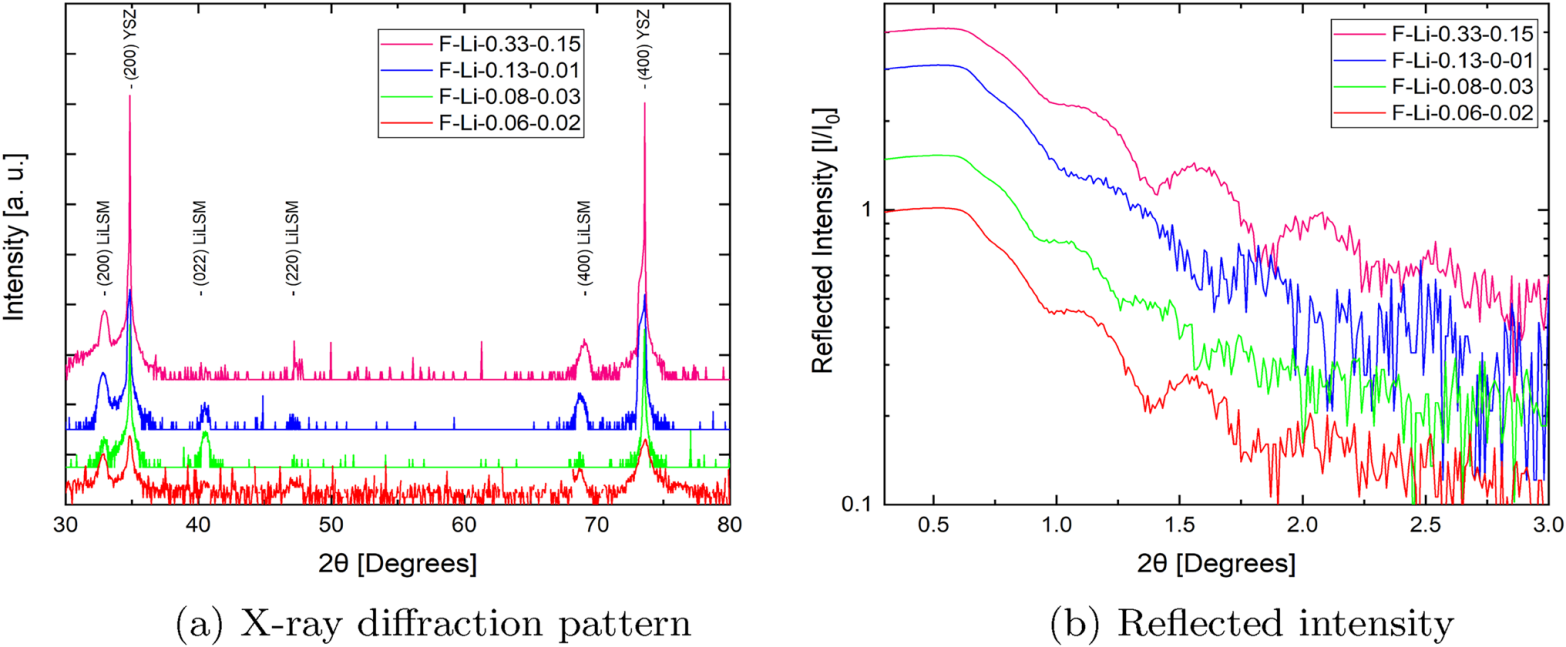
**a** X-ray diffraction patterns of thin films ablated from different targets using the same process parameters, showing the (200), (022), (200), (400) film and (200), (400) YSZ substrate orientation. The peaks for the films were indexed from [27] and the substrate peaks from [31]. **b** Reflected intensity (not corrected for footprint effects) of thin films ablated from the different targets using the same process parameters each, showing different oscillation lengths and therefore thicknesses

The structural analysis of the thin films grown at 923 K revealed a single crystalline structure (see Fig. 1a) oriented along the (h00) plane. The films exhibit the main film peak at a lower angle relative to the substrate, which is related to a larger lattice parameter. All the targets used to grow thin films for this study contained several phases. However, as it has been shown, the use of phase pure/single crystalline targets is not strictly mandatory for PLD.

The films grown at room temperature exhibited an X-ray amorphous structure.

X-ray reflectivity data (see Fig. 1b) were recorded to calibrate the growth rate under the same process parameters. Interestingly, films F-Li-0.33-0.15 and F-Li-0.13-0.01 showed an average growth of 0.125 Å per laser pulse and films made from targets containing less lithium, i.e. F-Li-0.08-0.03 and F-Li-0.06-0.02 grew with 0.171  Å per pulse. This variation in growth rate could be due to differences in target density and therefore absorption coefficient. [32]

**Fig. 2:**
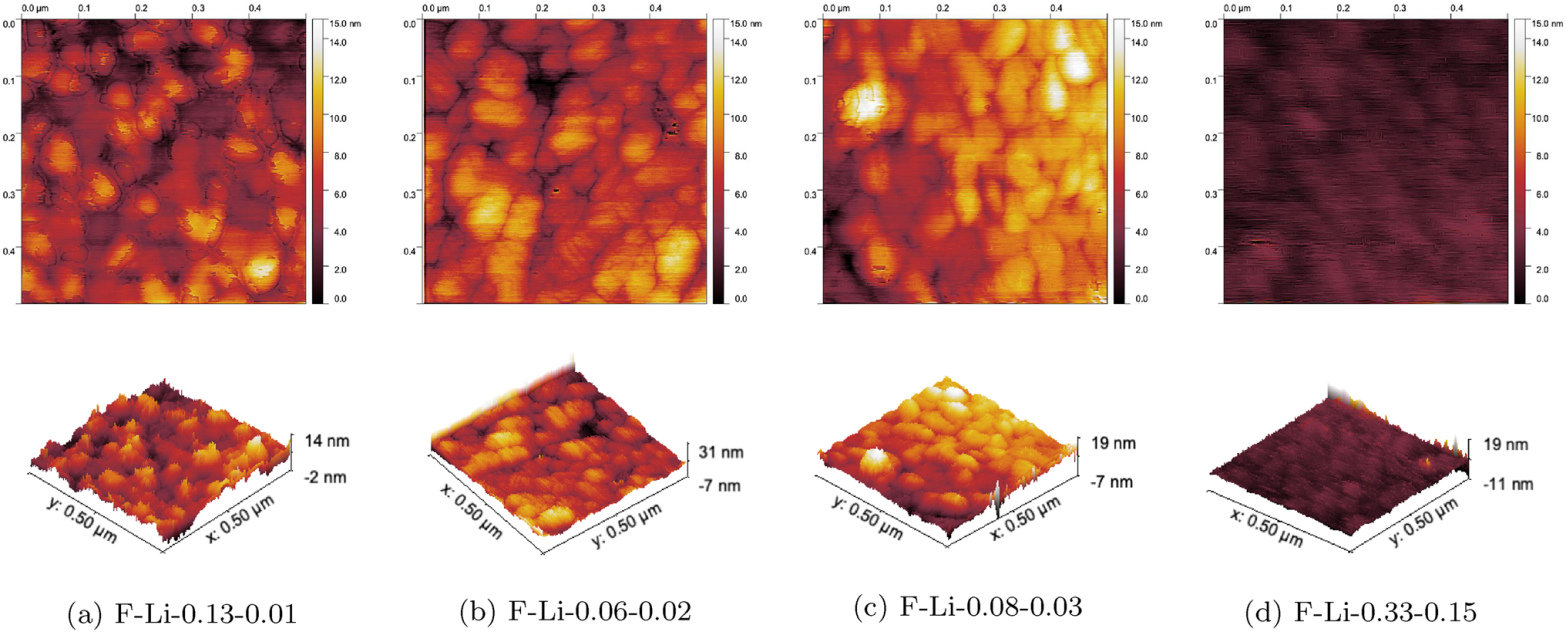
Height maps and surface reliefs obtained by AFM of F-Li-0.13-0.01 **a**, F-Li-0.06-0.02 **b**, F-Li-0.08-0.03 **c** and F-Li-0.33-0.15 **d** grown at 923 K, indicating a decrease in surface roughness and grain size for increasing lithium content

**Fig. 3:**
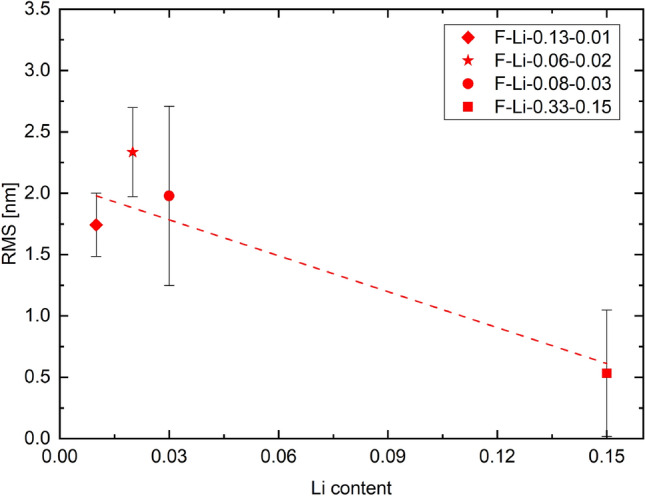
Root-mean-square roughness of F-Li-0.13-0.01, F-Li-0.06-0.02, F-Li-0.08-0.03 and F-Li-0.33-0.15 grown at 923 K. The dashed line acts as a guide to the eye since the RMS for the first three films lie within their error bars

Height maps and surface reliefs obtained by AFM measurements for the films grown at 923K are shown in Fig. 2. The root-mean-square (RMS) surface roughness was evaluated as 1.74, 2.34, 1.98 and 0.53 nm for rising lithium content (see Fig. 3) and a decrease in surface roughness and estimated grain size is hinted. These images indicate smooth and homogeneous films for higher lithium content. [33, 34]

#### Composition

For the composition, several PLD process parameters are coming into play:

Near the target we can assume a similar average kinetic energy of the same ablated species for all targets, since their kinetic energy is related to the ablation process and the laser fluence which was kept constant at 1.5 J/cm^2^. However, the kinetic energy is also a function of the species’ mass and is additionally influenced by the used background gas, pressure and interactions within the plasma plume.

The plasma plume travels to the substrate in a few μs. Depending on the pressure and type of background gas the ablated species may encounter background gas species and interact elastically or react. The background gas (in our case O_2_) is not only used to incorporate elements into the film but also to control the kinetic energy of the arriving species. Generally, there are electrons, positive and negative ions plus neutrals in the generated plasma. These species can be excited or in ground states, additionally there can be diatomics [35] and clusters. [36] Preferentially, metals form diatomics with oxygen. The concentration of those species is proportional to their stability, meaning their bond dissociation energy, i.e. whether this is larger or smaller than the dissociation energy of oxygen of 5.2 eV. Amoruso et. al [37] classified the pressure regime $p_{{\mathrm{O}}_{2}}$ = 10 Pa *diffusion-like* with a very slow expansion of the plume and a broad angular distribution of the ablated species. In the *diffusion-like* regime, the background gas confines all species together in a very slow expanding plume with all species travelling at a similar velocity. The mean free path (MFP) $\lambda$ of the species (see Table 3) is largely influenced by the pressure regime used for a given background gas. If the MFP is smaller than a tenth of the target-to-substrate distance, Amoruso et. al [37] defined this pressure regime as the *transition* regime which already allows chemical reactions between the background gas and the plasma plume while the plume species still have a relatively high kinetic energy when arriving at the substrate.

To achieve the formation of a crystalline LiLSM lattice, a high temperature of the substrate is needed. Heating of the substrate can have an influence on the composition and growth mode of the film. [38] In vacuum, the substrate temperature does not have a direct influence on how the species arrive at the substrate but only once they arrive. [39] However, when using a background gas the high substrate temperature heats the background gas, creating a gas density gradient which then affects the expansion dynamics of the plasma plume. This means that variations of the substrate temperatures affect the amount of gas species in the path of the plume expansion and therefore varying the interaction between the plume and the background gas. Higher substrate temperatures at a given pressure will result in plume expansion dynamics typical of that at a lower pressure and vice versa. Hence, this variation of the background pressure shifts the deposition regime.

Another aspect of temperature at the substrate is re-evaporation, e.g. for Li at *T* greater than 773 K a high loss was reported by F. Simmen. [40]

However, the most critical influence that the selected target material may have on the deposited films is their composition. For multi-element target materials, Ojeda et. al [10] reported a strong relationship between the atomic mass ratios of the target elements and the compositional deviations in the film. E.g. for LiMn_2_O_4_ with a mass ratio of Li to Mn = 1:8, pressure dependent deviations up to 70% were observed. The deviations may be even higher if heavier elements are applied, (here La, Sr) with a maximum mass ratio of Li to La = 1:20. During this study, deviations in lithium content of up to 90% were observed (F-Li-0.13-0.01). Lanthanum and strontium were transferred congruently.

**Distance variation**: For these experiments, the oxygen background pressure (10 Pa), temperature (923 K), laser fluence and target material (T-Li-0.13) were kept constant but the target-to-substrate distance was varied. For a distance of 4 cm, a lithium content of 0.01 was observed. At a distance of 7 cm, the lithium content was 0.1. This enrichment of lithium for the larger distance could be due to the change from the *diffusion-like* (at 4 cm) to the *transition* pressure regime, since the MFP (see Table 3) of the species would be below a tenth than the target-to-substrate distance as mentioned above. Since this regime allows for more chemical reactions between the species in the background gas and the formation of $\mathrm{Li-O}^{+}$ might be favoured on the cost of $\mathrm{Li}^{+}$ in comparison to the *diffusion-like* regime. At a distance of 4 cm, more $\mathrm{Li}^{+}$ than $\mathrm{Li-O}^{+}$ species might arrive at the heated substrate. Therefore, stronger lithium loss is observed, since the vapour pressure of lithium is 8 orders of magnitudes higher than for bound lithium in e.g. Li_2_O. [41]

**Table 3:** MFP of the elements contained in the used targets and thin films for 10 and $10^{-3}$ Pa oxygen base pressure. Calculated according to $\lambda = \frac{k_{\textit{B}} \times T}{\sqrt{2} \times P \times \pi \times d_{m}^{2}}$ with $k_{{B}}$ being the Boltzmann-constant, *T* the temperature of the gas, *P* the pressure and $d_{m} = (d_{O_{2}}) + d_{species}/2$, *d* diameter of the species

Element	Mass	MFP in $O_{2}$ (10 Pa)	MFP in $O_{2}$ ($10^{-3}$ Pa)
	[amu]	[mm]	[mm]
Lithium	6.9	2.84	$2.84 \times 10^{5}$
Strontium	87.6	2.06	$2.06 \times 10^{5}$
Lanthanum	138.9	2.12	$2.12 \times 10^{5}$
Manganese	54.9	2.93	$2.93 \times 10^{5}$
Oxygen	16.0	5.28	$5.28 \times 10^{5}$

**Fig. 4:**
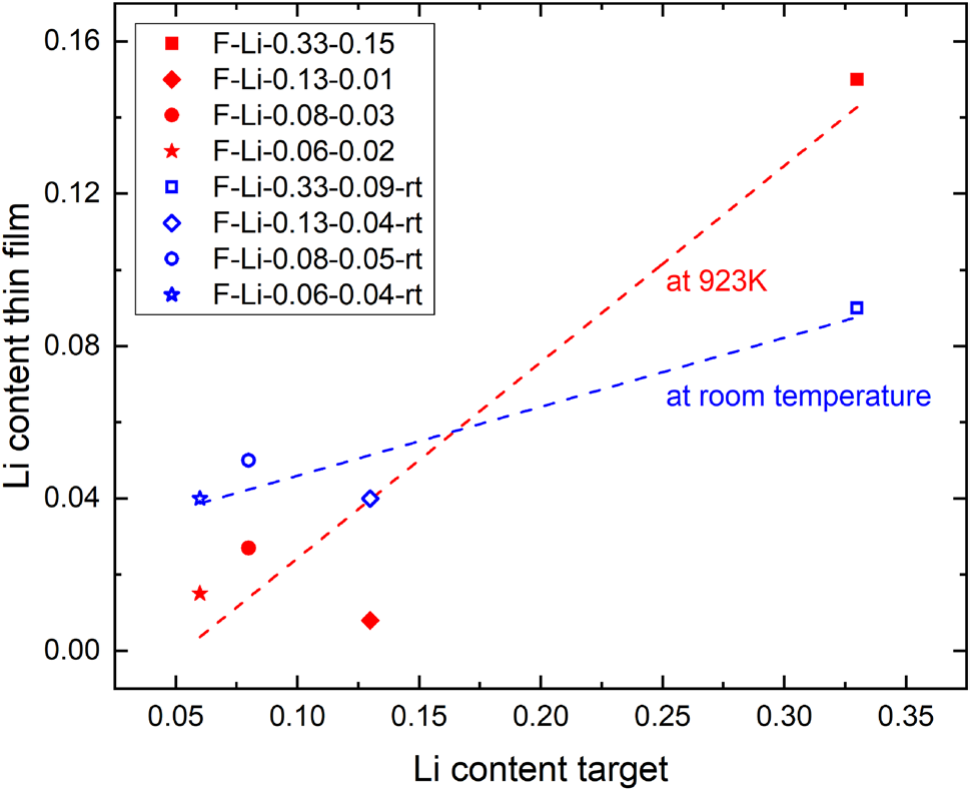
Lithium content (pfu) in the targets and thin films (relative to Mn=1) for ablations at 923 K and at room temperature, the dashed lines are guides for the eyes

**Temperature variation**: Another series of experiments comparing compositions at 923 K and at room temperature (see Fig. 4) revealed that for the targets with lower lithium content, namely F-Li-0.13, F-Li-0.08, F-Li-0.06, the lithium content was higher for the room temperature ablations than for the ones at 923 K. At room temperature, 4 cm target-substrate distance and $p_{O_{2}}$ $\approx$ 10 Pa the pressure regime for the ablation is diffusion-like, meaning that all species are in a confined plasma plume and will arrive at the substrate at the same time. The evaporation of lithium from the unheated substrate is also much lower. Therefore, more lithium will end up in the film at room temperature compared to higher temperatures (923 K).

Interestingly, for target F-Li-0.33, the lithium content in the film is higher than for the room temperature ablation. This effect has to be investigated further.

After observing all these effects of parameter variation on the composition of the thin films, the right combination of target-to-substrate distance, background pressure and growth temperature was chosen to achieve the desired sample.

## Conclusion

Four LiLSM PLD targets were synthesised using the solid-state route. The compositional analysis showed that one can compensate for the loss of lithium during sintering by enriching the powder with lithium before the last sintering step in agreement with literature.

Phase analysis indicated that all targets contained multiple phases while the thin films grown at 923K are all single-phased.

This thin film growth study confirmed that loss of lithium growing multi-element oxides with PLD is inevitable but to some extent can be controlled by choosing the right growth conditions as temperature, background pressure, stabilised phases and especially target-to-substrate distance.

During this study, we were able to find the process parameters to grow epitaxial LiLSM thin films with the planned composition. Namely, a lithium content of 0.10 by using a target with 30% excess lithium (relative to the aimed for composition), a substrate temperature of 923K, a target-to-substrate distance of 7 cm and an oxygen background pressure of 20 Pa. Further studies investigating magnetic and other physical properties will be conducted with these samples.
